# Platelet-to-high-density lipoprotein cholesterol ratio as a predictor of stroke risk: a longitudinal analysis of the China health and retirement longitudinal study

**DOI:** 10.3389/fneur.2025.1503743

**Published:** 2025-09-16

**Authors:** Qilin Feng, Liuying Ren, Sijiang Chen, Xiaoqiang Li

**Affiliations:** ^1^Department of Neurology, Panzhou People’s Hospital, Panzhou, Guizhou, China; ^2^Department of Neurology, Xiaolan People’s Hospital of Zhongshan (The Fifth People’s Hospital of ZhongShan), Guangdong, China

**Keywords:** platelet-to-high-density lipoprotein cholesterol ratio, stroke, risk prediction, longitudinal study, CHARLS, China

## Abstract

**Background:**

Stroke is a major health concern in aging populations. This study investigates the platelet-to-high-density lipoprotein cholesterol ratio (PHR) as a predictor of stroke risk in middle-aged and older Chinese adults.

**Methods:**

We analyzed data from 8,405 participants aged ≥45 years in the China Health and Retirement Longitudinal Study (CHARLS), with an 8-year follow-up. PHR was calculated from baseline blood samples. Multivariate logistic regression models, supplemented by smooth curve fitting to assess dose–response relationships and subgroup analyses with interaction testing, assessed the association between PHR and stroke incidence, adjusting for various factors.

**Results:**

During follow-up, 753 (8.96%) participants reported a stroke. Smooth curve fitting visually confirmed a linear dose–response relationship between PHR and stroke probability. Each standard deviation increase in PHR was associated with 17% higher odds of stroke (OR: 1.17; 95% CI: 1.08–1.26, *p* < 0.05). The highest PHR quartile had 42% higher odds of stroke compared to the lowest (OR: 1.42; 95% CI: 1.14–1.76, *p* = 0.002), with a significant trend across quartiles (*p* < 0.05).

**Conclusion:**

Elevated PHR is independently associated with increased stroke risk in middle-aged and older Chinese adults. Incorporating PHR into existing risk assessment tools may enhance stroke risk stratification and guide preventive strategies. Further research is needed to elucidate underlying mechanisms and validate findings in diverse populations.

## Introduction

Stroke, characterized by sudden disruption of blood supply to the brain, remains a formidable challenge in global health, ranking among the leading causes of mortality and long-term disability worldwide (1–3). Its etiology is multifaceted, involving traditional risk factors such as hypertension, cardiac diseases, and diabetes mellitus, as well as the cumulative effect of vascular risk factors over time (4, 5). The severe and often irreversible consequences of stroke emphasize the critical need for enhanced preventive strategies and early risk assessment tools. In this context, the search for reliable and easily measurable biomarkers has intensified, aiming to improve risk stratification and enable timely interventions.

The PHR has emerged as a promising biomarker in recent cardiovascular and metabolic health research (6, 7), prompting further investigation into its clinical utility, particularly for complex vascular conditions like stroke. This ratio reflects the balance between pro-thrombotic and anti-thrombotic forces within the body, integrating two critical components implicated in cardiovascular health: platelets, which play a crucial role in thrombosis, and high-density lipoprotein cholesterol (HDL-C), known for its anti-atherogenic properties (8–10). Specifically, platelets actively participate in atherothrombosis, a primary driver of ischemic stroke, by adhering to disrupted atherosclerotic plaques, aggregating to form occlusive thrombi in cerebral arteries, and releasing mediators that promote vascular inflammation and plaque progression (11). Conversely, HDL-C offers multifaceted protection against cerebrovascular events by promoting reverse cholesterol transport from arterial walls, exerting crucial anti-inflammatory and antioxidant functions, and supporting endothelial health, thereby mitigating atherogenesis (12). Given these contrasting pathophysiological roles, the PHR is hypothesized to offer a more holistic reflection of the pro-thrombotic versus anti-thrombotic balance critical in stroke development than either marker in isolation. Therefore, an elevated PHR, potentially signifying both increased pro-thrombotic potential and diminished vasoprotective capacity, might reflect a heightened underlying vulnerability to stroke development. Recent studies have expanded our understanding of PHR’s potential applications, linking it to various aspects of vascular health, metabolic syndrome, and cardiovascular risk (7, 13). Notably, research by Zhang et al. (2024) revealed a significant association between elevated PHR and increased risk of both self-reported stroke and cardiovascular mortality, underscoring its potential utility in identifying individuals at higher risk of stroke and cardiovascular events (14).

Despite these promising developments, there remains a notable gap in the literature, particularly concerning the Chinese population. China faces a unique challenge with its rapidly aging population and a rising prevalence of stroke among the elderly (15, 16). The relative scarcity of evidence from Chinese cohorts, compared to Western populations, highlights an urgent need for focused research in this demographic. Our study aims to address this critical gap by investigating the relationship between PHR and stroke risk in a large Chinese cohort, utilizing data from the China Health and Retirement Longitudinal Study. By exploring the predictive value of PHR in this context, we seek to provide clinically relevant evidence that could inform stroke prevention and management strategies tailored to the Chinese population, ultimately contributing to the development of more effective risk assessment tools and improved outcomes for individuals at risk of stroke in China and beyond.

## Materials and methods

### Study design

In this prospective cohort study, we utilized data from the China Health and Retirement Longitudinal Study (CHARLS) collected between 2011 and 2020 (17). CHARLS is a nationally representative survey assessing the health and socioeconomic status of middle-aged and older Chinese adults aged 45 years and older (17). In this study, the PHR was investigated as the primary exposure variable. The primary outcome was incident stroke during the follow-up period. This prospective design enabled the longitudinal analysis of the association between baseline PHR and subsequent stroke risk in this population.

### Study population and data source

The study cohort was selected from the publicly available CHARLS database. CHARLS was initially designed to provide a robust foundation for scientific research on China’s rapidly aging population, encompassing residents aged 45 years and above (13). The initial baseline survey, conducted in 2011, enrolled 17,705 participants across a diverse geographic spread, covering 28 provinces, 150 counties/districts, and 450 villages/urban communities. This extensive sampling strategy aims to ensure a comprehensive representation of China’s demographic and socioeconomic diversity.

Subsequent follow-up surveys were conducted in 2013–2014 (wave 2), 2015–2016 (wave 3), 2017–2018 (wave 4), and 2019–2020 (wave 5), providing longitudinal data. Blood samples were collected during wave 1 (baseline) and wave 3. For this study, we focused on participants from the 2011 baseline cohort and utilized blood sample data collected during this initial wave. These individuals were then followed prospectively through waves 2, 3, 4, and 5 to monitor the incidence of stroke.

### Participant selection

**Figure 1:**
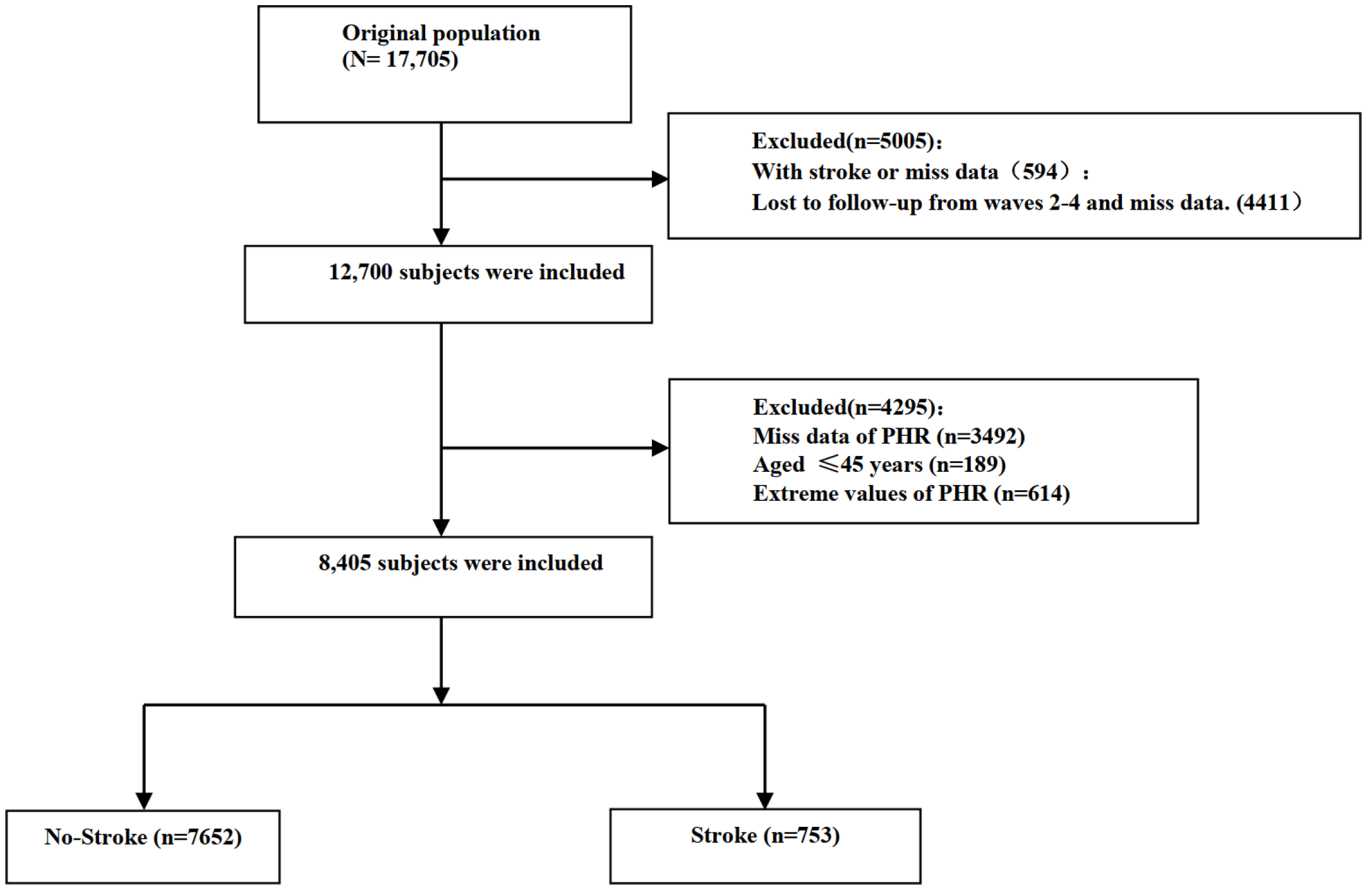
Flow chart visualizing the patient selection process.

From the initial 17,705 participants in the baseline survey of CHARLS, we applied several exclusion criteria to construct the analytical cohort. First, we excluded participants who reported a history of stroke at baseline or had missing baseline information relevant to stroke assessment (*n* = 594). Additionally, we excluded individuals who were completely lost to follow-up (i.e., provided no data in waves 2, 3, 4, or 5) or had missing stroke outcome data during follow-up (*n* = 4,411). After this initial screening, 12,700 participants remained. Subsequently, we further excluded participants with missing baseline PHR data (*n* = 3,492) and those aged less than 45 years (*n* = 189). Finally, to mitigate the potential undue influence of extreme values, participants with extreme PHR values were excluded based on the mean ± 3 standard deviations (SD) threshold. Applying this criterion, participants with PHR values greater than 398.091 were defined as outliers and excluded (*n* = 614). This approach to outlier handling has been utilized in other health-related studies (18). Following all exclusion steps, a final sample of 8,405 participants was included in the longitudinal analysis. The detailed participant selection process is illustrated in Figure 1.

### Clinical variables and laboratory variables

Our study incorporated a comprehensive set of demographic and clinical variables extracted from the CHARLS database. These included age, sex, anthropometric measurements (height, weight, body mass index), lifestyle factors (marital status, smoking and drinking habits), and pertinent medical history (diabetes, hypertension, heart disease, kidney disease). This extensive profiling allowed for a thorough characterization of our study population and potential confounding factors.

The laboratory assessment encompassed a wide array of biomarkers crucial for cardiovascular and metabolic health evaluation. We also analyzed lipid profiles (low-density lipoprotein cholesterol, high-density lipoprotein cholesterol, triglycerides, total cholesterol). The PHR, our primary variable of interest, was calculated by dividing the platelet count by HDL-C levels (14, 19), following established methodologies in cardiovascular research.

### Diagnosis of stroke

The identification of stroke events in our cohort was based on a comprehensive follow-up protocol designed to capture incident cases through self-reported physician diagnoses. Participants who were stroke-free at baseline but reported a physician-diagnosed stroke during subsequent follow-ups were classified as having experienced a stroke event. This classification relied on standardized questionnaires administered during each follow-up wave, which specifically inquired about: (i) whether participants were told by a doctor that they had been diagnosed with a stroke, (ii) the timing of diagnosis, and (iii) whether they were currently receiving any follow-up treatment for their stroke. Affirmative responses to these questions during any follow-up wave resulted in the participant being categorized into the stroke group. To maintain the integrity of our analysis, participants who did not report a stroke in any follow-up survey were classified as the healthy group, while individuals with no follow-up data were excluded from the final analysis to ensure the robustness of our longitudinal assessment. This self-reported physician diagnosis approach is commonly employed in large-scale epidemiological studies and has been validated in previous research (20, 21).

### Statistical analysis

Our statistical approach was designed to comprehensively examine the relationship between PHR levels and stroke incidence. Continuous variables were characterized using means and standard deviations for normally distributed data, or medians and interquartile ranges for non-normally distributed data, with comparisons between groups conducted using t-tests or Mann–Whitney U tests, as appropriate. Categorical variables were expressed as percentages and compared using chi-square tests or Fisher’s exact tests, depending on cell frequencies. To validate the linear relationship between PHR levels and stroke incidence, we employed curve fitting techniques. The association between PHR levels and stroke risk was then assessed through a series of regression analyses, progressively adjusting for potential confounders: Crude model (unadjusted), Model 1 (adjusted for age and sex), Model 2 (fully adjusted model 1 incorporating marital status, residence, education, smoking, drinking) and Model 3 (fully adjusted model 2 incorporating hypertension, dyslipidemia, diabetes, heart problems, kidney disease). Statistical significance was defined as *p* < 0.05 for all analyses. Data processing and statistical analyses were performed using R statistical software (version R 4.4.1, The R Foundation).

## Results

### Baseline characteristics

Our study cohort comprised 8,405 participants (mean age 57.93 ± 8.57 years, 55.11% female), stratified into quartiles based on their PHR (Table 1). Significant differences were observed across PHR quartiles for several cardiovascular risk factors. Body Mass Index increased from 22.61 ± 3.63 kg/m^2^ in Q1 to 24.73 ± 3.8 kg/m^2^ in Q4 (*p* < 0.05), while HDL-cholesterol levels decreased from 1.65 ± 0.41 mmoL/L to 1.01 ± 0.22 mmoL/L (*p* < 0.05). The prevalence of stroke showed an upward trend across PHR quartiles, rising from 8.33% in Q1 to 11.13% in Q4 (*p* < 0.05). Similarly, the prevalence of hypertension, diabetes, and dyslipidemia increased significantly with higher PHR quartiles (all *p* < 0.05). These baseline characteristics demonstrate significant associations between PHR levels and various cardiovascular risk factors in the study population. Supplementary Table 1 presents the baseline characteristics of participants according to the occurrence of stroke during the follow-up period. Participants who developed stroke were significantly older, had higher baseline PHR values, higher BMI, lower HDL-cholesterol levels, and were more likely to have hypertension, diabetes, and dyslipidemia compared to those who remained stroke-free (all *p* < 0.05).

**Table 1:** Characteristics of participants.

Characteristics	Total (n = 8,405)	Q1 (<116.9)	Q2 (116.9–159.9)	Q3 (159.9–213.8)	Q4 (>213.8)	*p*	SMD
No.	8,405	2,101	2,102	2,100	2,102		
Age	57.93 ± 8.57	59.32 ± 8.89	57.99 ± 8.45	57.29 ± 8.35	57.11 ± 8.41	<0.05	0.14
Sex, (%)						0.05	0.05
Female	4,632 (55.11)	1,116 (53.12)	1,142 (54.33)	1,173 (55.86)	1,201 (57.14)		
Male	3,773 (44.89)	985 (46.88)	960 (45.67)	927 (44.14)	901 (42.86)		
Residence						<0.05	0.13
rural	5,550 (66.03)	1,494 (71.11)	1,426 (67.84)	1,365 (65.00)	1,265 (60.18)		
urban	2,855 (33.97)	607 (28.89)	676 (32.16)	735 (35.00)	837 (39.82)		
Smoking						0.06	0.04
No	6,037 (71.83)	1,498 (71.30)	1,483 (70.55)	1,499 (71.38)	1,557 (74.07)		
Yes	2,368 (28.17)	603 (28.70)	619 (29.45)	601 (28.62)	545 (25.93)		
Education						<0.05	0.13
College or above	120 (1.43)	20 (0.95)	35 (1.67)	30 (1.43)	35 (1.67)		
Middle school	2,535 (30.16)	525 (24.99)	634 (30.16)	653 (31.10)	723 (34.40)		
No formal education	3,879 (46.15)	1,092 (51.98)	961 (45.72)	918 (43.71)	908 (43.20)		
Primary school	1871 (22.26)	464 (22.08)	472 (22.45)	499 (23.76)	436 (20.74)		
Drinking						<0.05	0.12
No	4,988 (59.35)	1,134 (53.97)	1,203 (57.23)	1,306 (62.19)	1,345 (63.99)		
Yes	3,417 (40.65)	967 (46.03)	899 (42.77)	794 (37.81)	757 (36.01)		
BMI (kg/m2)	23.61 ± 3.8	22.61 ± 3.63	23.08 ± 3.52	24.01 ± 3.88	24.73 ± 3.8	<0.05	0.33
TC (mmol/L)	5.01 ± 0.98	5.04 ± 0.92	5.02 ± 0.95	5.02 ± 0.98	4.98 ± 1.06	0.24	0.03
HDL(mmol/L)	1.33 ± 0.39	1.65 ± 0.41	1.41 ± 0.31	1.24 ± 0.26	1.01 ± 0.22	<0.05	1.15
LDL(mmol/L)	3.03 ± 0.89	2.94 ± 0.81	3.06 ± 0.87	3.11 ± 0.88	2.99 ± 0.98	<0.05	0.11
TG(mmol/L)	1.48 ± 1.11	1.11 ± 0.64	1.28 ± 0.83	1.5 ± 0.91	2.04 ± 1.58	<0.05	0.46
Stroke, (%)	753 (8.96)	175 (8.33)	152 (7.23)	192 (9.14)	234 (11.13)	<0.05	0.07
Hypertension, (%)	1969 (23.43)	438 (20.85)	424 (20.17)	519 (24.71)	588 (27.97)	<0.05	0.11
Diabetes, (%)	421 (5.01)	77 (3.66)	88 (4.19)	115 (5.48)	141 (6.71)	<0.05	0.08
Dyslipidemia, (%)	751 (8.94)	130 (6.19)	150 (7.14)	186 (8.86)	285 (13.56)	<0.05	0.14
Heart Problems, (%)	918 (10.92)	215 (10.23)	205 (9.75)	237 (11.29)	261 (12.42)	0.03	0.05

### Relationship between PHR and stroke probability

**Figure 2:**
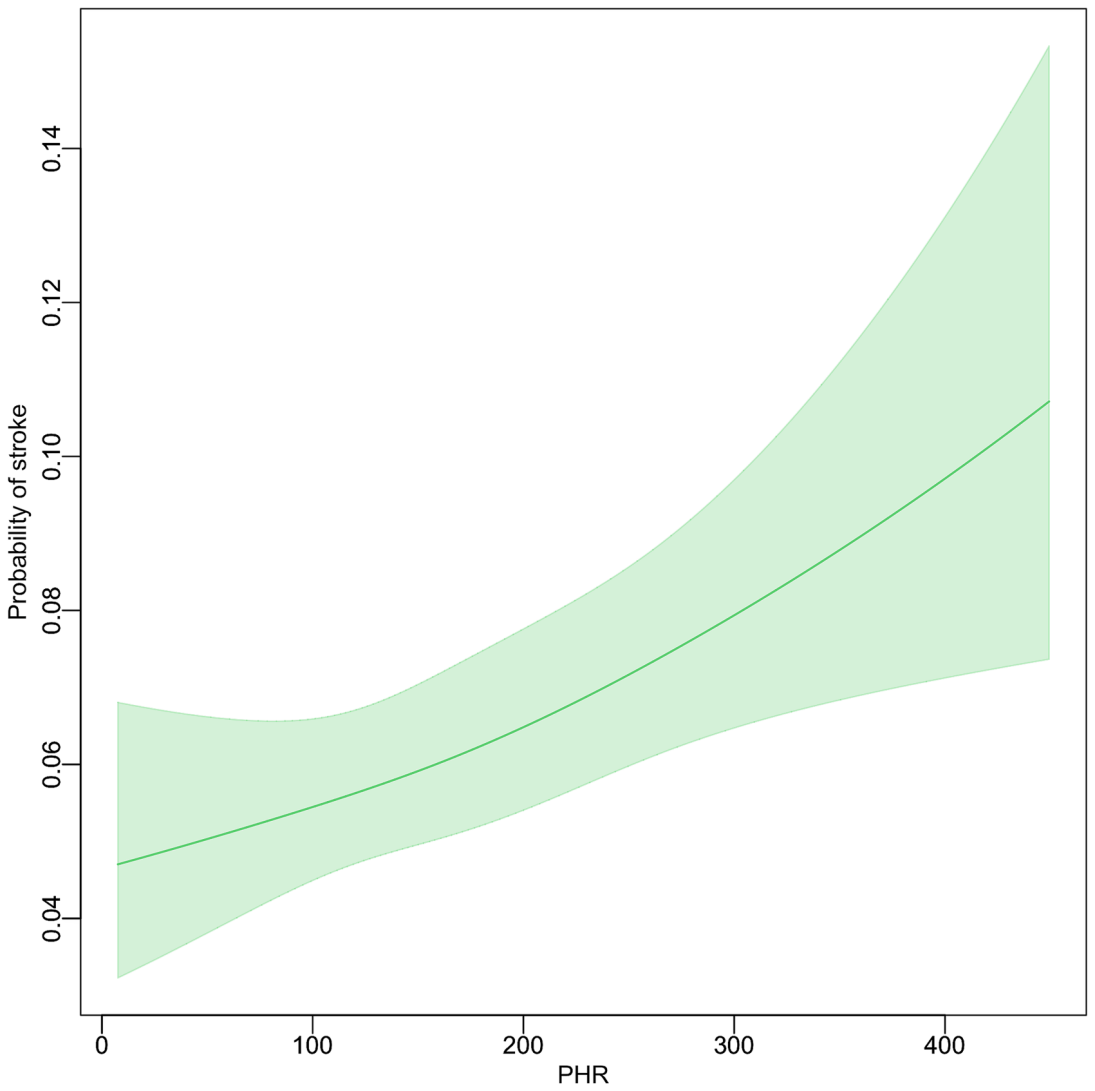
The relationship between PHR level and the incidence of stroke.

The association between the PHR and the probability of stroke was examined using smooth curve fitting, after adjusting for multiple variables (Figure 2). The adjustments included sex, age, marital status, residence, education, smoking, drinking, hypertension, dyslipidemia, diabetes, heart problems, and kidney disease. The resulting graph shows a linear relationship between PHR and stroke probability. As PHR values increase, there is a corresponding increase in the probability of stroke. The 95% confidence interval, represented by the shaded area around the line, remains relatively narrow throughout the PHR range, widening slightly at higher PHR values.

### Multivariate analysis of the relationship between PHR and stroke

The association between PHR and stroke risk was examined using multiple logistic regression models. In the crude model, each standard deviation increase in PHR was associated with a 17% higher odds of stroke (OR = 1.17, 95% CI: 1.09–1.25, *p* < 0.05). After adjusting for sex and age (Model 1), the association remained consistent (OR = 1.21, 95% CI: 1.12–1.30, *p* < 0.05). Model 2, which included additional adjustments, showed similar results to Model 1 (OR = 1.21, 95% CI: 1.12–1.30, *p* < 0.05). In the fully adjusted model (Model 3), which controlled for multiple variables, the association persisted (OR = 1.17, 95% CI: 1.08–1.26, *p* < 0.05). When PHR was analyzed by quartiles, the highest quartile (Q4) consistently showed a significantly higher odds of stroke compared to the lowest quartile (Q1) across all models. In the fully adjusted model, participants in Q4 had 42% higher odds of stroke compared to those in Q1 (OR = 1.42, 95% CI: 1.14–1.76, *p* = 0.002). A significant trend was observed across PHR quartiles in all models (*p* for trend < 0.05), indicating a dose–response relationship between PHR levels and stroke risk. The continuous and quartile-based analyses showed consistent patterns, with both approaches demonstrating significant positive associations between PHR and stroke risk.

### Subgroup analyses and interaction effects

**Figure 3:**
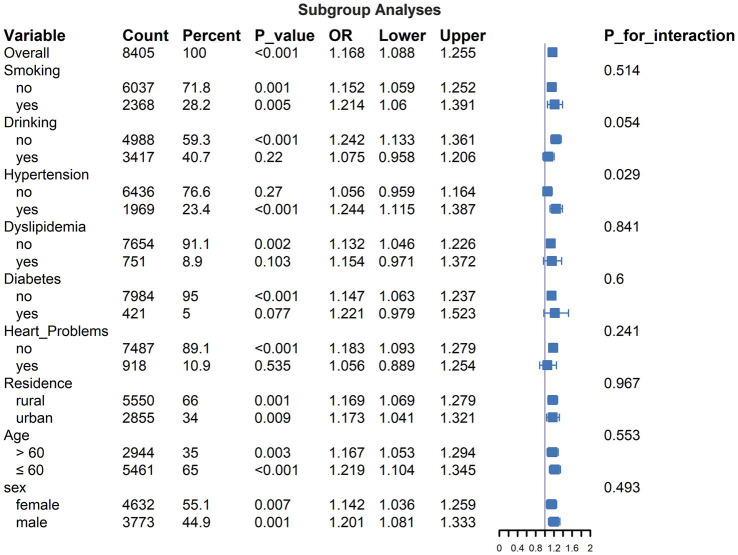
Subgroup analyses for the association between PHR (PHR, per standard deviation increase) and incident stroke.

Subgroup analyses examining the association between PHR (per standard deviation increase) and incident stroke are detailed in Figure 3. The overall adjusted OR was 1.168 (95% CI: 1.088–1.255, *p* < 0.05). Within strata, the association did not reach statistical significance (*p* ≥ 0.05) among participants reporting current drinking, those without baseline hypertension, or those with baseline dyslipidemia, diabetes, or heart problems. The test for interaction between PHR and baseline hypertension status resulted in a *p* value of 0.029. All other *p* -values for tested interactions were ≥0.054.

## Discussion

In this retrospective cohort study, we investigated the association between the PHR and the likelihood of stroke occurrence. Analyzing data from 8,405 middle-aged and elderly Chinese adults aged 45 and above, our study leverages a substantial sample size and an extensive follow-up period. Our findings reveal a positive linear relationship between PHR and the incidence of stroke. Specifically, we observed that an increase in PHR is significantly associated with a higher risk of stroke, with this relationship persisting across various adjustment models. In the fully adjusted model, for every one standard deviation increase in PHR, there was a 17% heightened odds of stroke (OR: 1.17; 95% CI: 1.08–1.26, *p* < 0.05) over the follow-up period. Furthermore, participants in the highest PHR quartile demonstrated 42% higher odds of stroke compared to those in the lowest quartile (OR: 1.42; 95% CI: 1.14–1.76, *p* = 0.002). A significant trend across PHR quartiles (*p* for trend <0.05) indicates a dose–response relationship between PHR levels and stroke risk. These findings underscore the potential importance of PHR as a marker for stroke risk assessment and highlight the need for further research into its utility as a predictive tool in clinical settings, particularly within the Chinese population.

Platelets contribute to inflammation, a significant factor in stroke development, by releasing mediators that exacerbate vascular inflammation and promote atherosclerosis and plaque rupture (22, 23). Interactions between platelets and leukocytes further create a thrombotic environment, contributing to stroke pathogenesis (24). Thus, an elevated platelet/HDL-C ratio may reflect a high inflammatory state, increasing stroke risk. Higher high-density lipoprotein cholesterol (HDL-C) levels are associated with a reduced risk of cardiovascular disease, including stroke (12). Dyslipidemia, marked by elevated LDL-C and reduced HDL-C, is strongly linked to coronary artery disease, atherosclerosis, and cerebrovascular disease (25). Thus, both platelet count and HDL-C levels are significant in atherosclerosis and cardiovascular disease. Investigating the clinical significance of the platelet-to-HDL ratio could reveal its value in predicting and managing cardiovascular and cerebrovascular diseases. Our study also demonstrated that this ratio predicts stroke incidence in patients.

Our findings align with Zhang et al. (14), who identified a significant association between the PHR and stroke risk as well as cardiovascular mortality in a retrospective study of 27,301 participants. They reported an OR of 1.20, while our study with 8,405 participants found an OR of 1.17 for stroke incidence over 8 years. While our study employed a prospective cohort design, Zhang et al. conducted a retrospective analysis. Zhang et al. observed a threshold effect (PHR ≥ 223.684) whereas we found a linear relationship, with stroke risk increasing progressively with higher PHR values. These differences may stem from variations in study design and population demographics, with our study focusing on stroke incidence in middle-aged and elderly Chinese individuals.

Lu et al. (26) explored the association between PHR and nonalcoholic fatty liver disease (NAFLD) using NHANES data in a cross-sectional study. Their findings support the use of PHR as a biomarker for NAFLD, but the cross-sectional design limits causal inference compared to our cohort study, which suggests a stronger cause-effect relationship. Ni et al. (27) found a similar predictive value of PHR for nephrolithiasis, further validating its broader applicability. Our study’s prospective focus on stroke incidence adds unique insights, highlighting the importance of PHR in predicting stroke risk specifically in an elderly Chinese cohort.

The clinical implications of our findings are substantial. Monitoring PHR could become a valuable tool in the early identification of individuals at heightened risk of stroke, allowing for timely and targeted preventive measures. This is particularly pertinent in aging populations where stroke prevalence is high and the burden of disease is significant. Incorporating PHR assessment into routine clinical practice could enhance risk stratification and guide intervention strategies, ultimately improving patient outcomes.

This study possesses several notable strengths. Firstly, our large sample size of 8,405 participants enhances the statistical power and reliability of our findings. Secondly, focusing on the middle-aged and elderly Chinese population provides significant clinical relevance, given the increasing aging population in China and the scarcity of evidence-based research in this demographic. Thirdly, employing curve fitting statistical methods allowed for a more nuanced visualization of the relationship between the PHR and stroke risk. Lastly, our study adjusted for a comprehensive range of covariates, including hypertension, diabetes, glycated hemoglobin, and lipid levels, ensuring robust and stable results. These methodological strengths collectively contribute to the credibility and applicability of our findings.

This study has several limitations. First, our findings are based on a cohort of middle-aged and elderly Chinese individuals, which may limit generalizability to other populations with different genetic, environmental, and lifestyle factors. Second, the CHARLS dataset lacks information on stroke subtypes and etiologies, precluding analysis of associations by stroke type or cause. Third, detailed data on specific antithrombotic or lipid-lowering medications, including drug names, dosages, and duration, were unavailable. This may have introduced residual confounding related to medication use. Fourth, our stroke case identification relied on self-reported physician diagnoses rather than medical record verification or clinical adjudication, which could potentially introduce misclassification bias. Finally, as an observational study, residual confounding cannot be completely excluded despite rigorous adjustment for potential confounders. Further studies in diverse populations are warranted to validate our findings.

## Conclusion

In conclusion, our study demonstrates a significant association between the PHR and stroke risk in an elderly Chinese population. The observed linear relationship indicates that as the PHR increases, the incidence of stroke becomes more pronounced. Given the ease of measuring PHR, our findings suggest that it has substantial clinical significance for stroke monitoring. These results underscore the importance of considering PHR in stroke risk assessment. Further research is necessary to validate these findings across diverse populations and through higher-level clinical studies.

## Data Availability

Publicly available datasets were analyzed in this study. This data can be found here: https://charls.pku.edu.cn/.

## Supplementary material

The Supplementary material for this article can befound online at: https://www.frontiersin.org/articles/10.3389/fneur.2025.1503743/full#supplementary-material
