# Supplementary material for: Platelet-to-high-density lipoprotein cholesterol ratio as a predictor of stroke risk: a longitudinal analysis of the China health and retirement longitudinal study
Source: Front Neurol. 2025 Sep 16;16:1503743. doi: 10.3389/fneur.2025.1503743 (PMC12479224; doi:10.3389/fneur.2025.1503743)
Supplement: Supplementary file 1 [file Table_1.docx]

Supplementary Table 1: Baseline characteristics by outcome status

| **Characteristics** | **Total** | **No-stroke** | **Stroke** | ***p*** |
| --- | --- | --- | --- | --- |
| **No.** | 8405 | 7652 | 753 |  |
| **age** | 57.93±8.57 | 57.63±8.52 | 60.96±8.52 | <0.05 |
| **Sex,(%)** |  |  |  | 0.79 |
| female | 4632 (55.11) | 4221 (55.16) | 411 (54.58) |  |
| male | 3773 (44.89) | 3431 (44.84) | 342 (45.42) |  |
| **Residence** |  |  |  | 0.92 |
| rural | 5550 (66.03) | 5051 (66.01) | 499 (66.27) |  |
| urban | 2855 (33.97) | 2601 (33.99) | 254 (33.73) |  |
| **Smoking** |  |  |  | 0.96 |
| no | 6037 (71.83) | 5495 (71.81) | 542 (71.98) |  |
| yes | 2368 (28.17) | 2157 (28.19) | 211 (28.02) |  |
| **Education** |  |  |  | 0.02 |
| College or above | 120 (1.43) | 115 (1.50) | 5 (0.66) |  |
| Middle school | 2535 (30.16) | 2334 (30.50) | 201 (26.69) |  |
| No formal education | 3879 (46.15) | 3520 (46.00) | 359 (47.68) |  |
| Primary school | 1871 (22.26) | 1683 (21.99) | 188 (24.97) |  |
| **Drinking** |  |  |  | 0.38 |
| no | 4988 (59.35) | 4553 (59.50) | 435 (57.77) |  |
| yes | 3417 (40.65) | 3099 (40.50) | 318 (42.23) |  |
| **BMI (kg/m2)** | 23.61±3.8 | 23.52±3.78 | 24.5±3.89 | <0.05 |
| **TC (mmol/L)** | 5.01±0.98 | 5±0.98 | 5.15±0.98 | <0.05 |
| **HDL(mmol/L)** | 1.33±0.39 | 1.33±0.39 | 1.28±0.38 | <0.05 |
| **LDL(mmol/L)** | 3.03±0.89 | 3.02±0.89 | 3.14±0.92 | <0.05 |
| **TG(mmol/L)** | 1.48±1.11 | 1.47±1.11 | 1.63±1.08 | <0.05 |
| **Hypertension, (%)** | 1969 (23.43) | 1656 (21.64) | 313 (41.57) | <0.05 |
| **Diabetes, (%)** | 421 (5.01) | 349 (4.56) | 72 (9.56) | <0.05 |
| **Dyslipidemia, (%)** | 751 (8.94) | 627 (8.19) | 124 (16.47) | <0.05 |
| **Heart Problems, (%)** | 918 (10.92) | 776 (10.14) | 142 (18.86) | <0.05 |
| **PHR** | 171.61±75.49 | 170.51±74.8 | 182.87±81.44 | <0.05 |
